# Real-world treatments and thrombotic events in polycythemia vera patients in the USA

**DOI:** 10.1007/s00277-023-05089-6

**Published:** 2023-01-13

**Authors:** Srdan Verstovsek, Naveen Pemmaraju, Nancy L. Reaven, Susan E. Funk, Tracy Woody, Frank Valone, Suneel Gupta

**Affiliations:** 1 MD Anderson Cancer Center, Houston, TX USA; 2 Strategic Health Resources, La Canada, CA USA; 3 Protagonist Therapeutics, Newark, CA USA

**Keywords:** Polycythemia vera, Myeloproliferative neoplasm, Thrombotic events, Hematocrit control, Therapeutic phlebotomy, Cytoreductive medication

## Abstract

Polycythemia vera (PV) is a myeloproliferative neoplasm associated with increased risk of thrombotic events (TE) and death. Therapeutic interventions, phlebotomy and cytoreductive medications, are targeted to maintain hematocrit levels < 45% to prevent adverse outcomes. This retrospective observational study examined medical and pharmacy claims of 28,306 PV patients initiating treatment for PV in a data period inclusive of 2011 to 2019. Study inclusion required ≥ 2 PV diagnosis codes in the full data period, at least 1 year of PV treatment history, and ≥ 1 prescription claim and medical claim in both 2018 and 2019. Patients having ≥ 2 hematocrit (HCT) test results in linked outpatient laboratory data (2018–2019) were designated as the HCT subgroup (*N* = 4246). Patients were characterized as high- or low-risk at treatment initiation based on age and prior thrombotic history. The majority of patients in both risk groups (60% of high-risk and 83% of low-risk) initiated treatment with phlebotomy monotherapy, and during a median follow-up period of 808 days, the vast majority (81% low-risk, 74% high-risk) maintained their original therapy during the follow-up period. Hematocrit control was suboptimal in both risk groups; 54% of high-risk patients initiating with phlebotomy monotherapy sometimes/always had HCT levels > 50%; among low-risk patients, 64% sometimes/always had HCT levels above 50%. Overall, 16% of individuals experienced at least 1 TE subsequent to treatment initiation, 20% (*n* = 3920) among high-risk and 8% (*n* = 629) among low-risk patients. This real-world study suggests that currently available PV treatments may not be used to full advantage.

**Supplementary Information:**

The online version contains supplementary material available at 10.1007/s00277-023-05089-6.

## Introduction

Polycythemia vera (PV) is a myeloproliferative neoplasm that is characterized by a primary, clonally driven abnormal increase in red blood cell mass. This overproduction of red blood cells is reflected in a patient’s elevated hematocrit (HCT) levels [1–4]. According to the World Health Organization (WHO) revised criteria of 2016, threshold HCT levels for the diagnosis of PV are > 49% for males and > 48% for females [4, 5], when combined with other diagnostic criteria. The estimated prevalence of PV is 45–57 cases per 100,000 persons in the USA [6].

Patients with PV have reduced survival compared to the age- and sex-matched population in the USA [3, 7], with a median survival ranging from 12.4 to 20 years [8, 9]. PV is associated with an increased risk of thrombotic or bleeding complications [6, 8, 10, 11], and thrombotic events represent the main cause of mortality for PV patients [12]. Annual incidence is positively associated with HCT levels and increases by age; in a meta-analysis of 16 studies (*n* = 3236 PV patients), thrombosis rates were 1.9%, 3.6%, and 6.8% per person-year at median ages 60, 70, and 80 years, respectively [13]. Bleeding events are less common, with a reported incidence of 1% per year [8].

Patients with PV are stratified into low-risk or high-risk categories according to age and previous history of thrombosis. Low-risk patients are defined as individuals younger than 60 years with no previous thrombosis, and high-risk patients are individuals older than 60 years and/or those with a history of thrombosis [9, 11].

HCT control is key to prevention of thrombotic events and other adverse outcomes [12, 14, 15]. The CYTO-PV study, an analysis that is central to clinical guidance in PV, reported that patients with a HCT target < 45% had a significantly lower rate of major thrombosis than did those patients with a HCT target of 45–50%. Moreover, in patients with PV who were receiving phlebotomy and/or hydroxyurea treatment, HCT maintenance of 45–50% was associated with four times the rate of death from cardiovascular causes or major thrombosis compared to patients with HCT maintenance < 45% [12].

Clinical guidelines recommend maintaining HCT < 45% for all patients with PV, regardless of risk status. For low-risk patients, newly revised NCCN guidelines recommend first-line treatment with phlebotomy to control HCT < 45% (phlebotomy should be done whenever HCT is above 45%), low-dose aspirin, and management of cardiovascular risk factors. Ropeginterferon alfa-2b-njft is an option for treatment of low-risk patients when the treating physician believes cytoreduction is needed [14]. For high-risk patients, cytoreductive therapy such as hydroxyurea, peginterferon alfa-2a, or ropeginterferon alfa-2b-njft is recommended first-line in addition to aspirin and management of cardiovascular risk factors. Ruxolitinib is recommended as a second line option. Phlebotomy should be used in high-risk patients as needed, but the goal of cytoreductive therapy is to eliminate a need for phlebotomy and to maintain HCT below 45% all the time.

In order to better understand real-world treatment patterns, we accessed insurance claims data to examine the patient journey among treated patients with PV, with specific reference to treatment initiation and the degree to which treatments changed over a relatively short period of time, and the association of patient risk status and the incidence of thrombotic events. In a subset of patients with available HCT data in a 2-year period, we also explored the degree of HCT management consistent with clinical guidelines, by risk status and type of PV treatment.

## Methods

We conducted a retrospective analysis of treatment pathways, frequency of TE outcomes, and HCT control following initiation of treatment for diagnosed PV using medical and pharmacy claims of US patients in the Symphony Health Solutions IDV® (Integrated Dataverse). The Symphony Dataverse includes de-identified longitudinal claims data for medical services and filled prescriptions on 280 million individuals in the USA and US territories from over 10,000 insurance plans offering Commercial, Medicare, and Medicaid coverage. Outpatient laboratory data from Quest Diagnostics was also available on approximately 35.7 million US adults in a dataset that is linked by patient identifier.

### Patient selection

Initial study selection required at least one diagnosis code for PV and at least one paid or approved claim for treatment of PV during calendar years 2018–2019. For patients meeting the initial criteria, medical claims from 2011 to 2019 were queried to find the first treatment for PV (index date) and retained for the assessment of treatments and outcomes. Eligible treatments included therapeutic phlebotomy and cytoreductive medications (hydroxyurea, ruxolitinib, busulfan, and relevant interferons). (see Online Resource 1 for detailed description of definitions and timelines.)

**Fig. 1:**
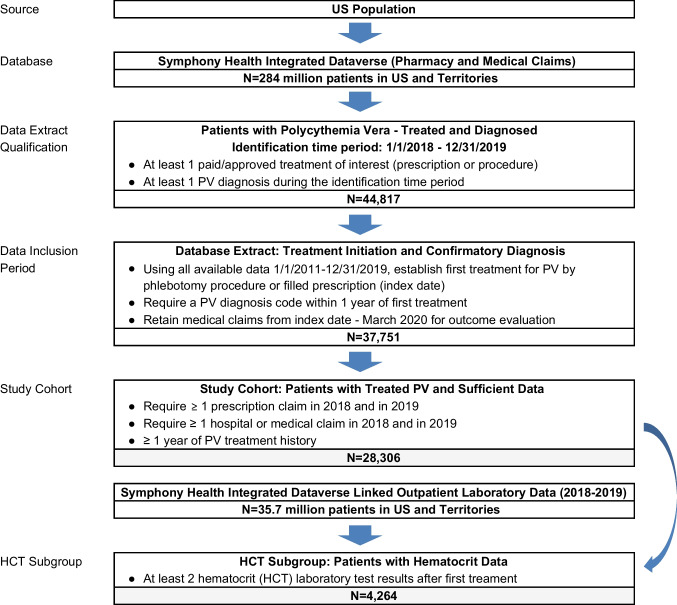
Patient selection flow chart

To qualify for the final study cohort, patients were required to have an additional PV diagnosis code within 1 year of the index date (for a total of at least two PV diagnosis codes in the full data period), at least 1 year of PV treatment history, and at least one prescription claim and one hospital or medical claim in both 2018 and 2019 (Fig. 1).

Patients having at least 2 hematocrit (HCT) test results post-index in linked outpatient laboratory data were designated as the HCT subgroup. Patients with ≥ 2 HCT tests averaged over 4.5 tests each.

### Patient characteristics and risk stratification

Age was evaluated as of the initial treatment for PV (index date). Race and ethnicity were derived from a combination of sources using Symphony’s proprietary methods and reported into the database. Prior thrombotic event was defined as the occurrence of a diagnosis code for thrombotic event (abdominal thrombosis, myocardial infarction, transient ischemic attack, stroke, pulmonary embolism, peripheral arterial thrombosis, deep vein, or other thrombosis or acute coronary syndrome) in any setting of care within the year prior to the index date (Online Resource 1).

Study cohort PV patients were stratified by the standard risk categories per National Comprehensive Cancer Network® (NCCN®) guidelines. [15] Patients aged ≥ 60 years at index or with a thrombotic event during the pre-index year were designated high-risk, while those under age 60 with no history of TE during the pre-index year were designated low-risk. Although a recent guideline update has refined recommended risk-stratification criteria [14], this study uses the earlier version that reflects criteria available to practicing physicians during the period of patient care profiled in this study.

### Therapeutic pathways

First-line therapy was defined as the therapeutic product or procedure first observed for the patient within 30 days of the index date, and for pharmaceutical agents, if repeated within 60 days post-index. First-line *combination* therapy was assigned if a claim for a pharmaceutical agent occurred within 30 days following an index phlebotomy treatment, or if a phlebotomy procedure or claim for a second drug was reported during the active period of a prescription (defined as remaining number of days-supply plus a 30-day grace period) for a drug prescribed at index.

Second and third therapy lines were identified at the next change in therapy by addition, switch, discontinuation, or restart. A new treatment was considered an *addition* if its first prescription fill or treatment date occurred within the active period of a prior therapy, and the prior therapy was repeated within the active period of the new treatment, irrespective of duration. A new treatment was considered a *switch* if the prior therapy was not repeated within the active period of the new treatment. *Discontinuation* was identified if a therapy was not repeated within its active period and the active period did not include the end of available data. *Restart* was identified if a discontinued therapy recurred in claims (Online Resource 1).

### Clinical outcomes

Thrombotic events were evaluated from treatment initiation through a data period extended to March 2020 during a median post-index period of 808 days, using all post-index claims data from January 2011 to March 2020. A thrombotic event was identified at the occurrence of a diagnosis code for a thrombotic event in medical claims data from any setting of care.

In the HCT subgroup, hematocrit control following treatment initiation was assessed among patients with at least two post-index HCT values in 2018 and 2019 by grouping HCT results by patient as always under 45%, under 50% but not always < 45%, sometimes over 50%, and always over 50%. The 45% cut point was selected to reflect clinical guidelines [15].

### Statistical analysis

Patient characteristics were compared between the high-risk and low-risk groups in both the full study cohort and the HCT subgroup using chi-square or unequal variance sample *t*-test as appropriate. Differences in characteristics between the full study cohort and the HCT subgroup were similarly evaluated.

Therapeutic pathways for the first, second, and third lines of therapy were summarized for the high-risk and low-risk groups, with therapies not involving either phlebotomy or hydroxyurea consolidated as “other”.

The number and percentage of patients experiencing at least one thrombotic event was evaluated for the high-risk and low-risk groups and within the high-risk group for patients with and without prior thrombotic events; these analyses were conducted in both the full study cohort and the HCT subgroup.

Hematocrit control following treatment initiation was assessed in the HCT subgroup, stratified by risk status, at three levels: in total, by first-line therapy, and by first and second therapy lines. Statistical evaluation of differences in HCT control between lines of therapy was performed using chi-square tests by comparing the proportion of patients in the always over 50% group (i.e., patients with all reported HCT values over 50%) and the always under 45% group, as clinical guidelines recommend. Pairwise comparisons were made between first-line therapies using patients initiating phlebotomy as the reference. A global comparison was made among all combinations of first- and second-line therapy. An additional global comparison was made among all therapy sequences involving both phlebotomy and hydroxyurea in any sequence or combination to assess whether HCT control differed significantly according to the sequence of these two therapies in patients receiving both.

Analyses were performed in Microsoft Excel and SAS/STAT® version 9.4 (Cary NC, USA). *p* values < 0.05 were considered statistically significant.

## Results

28,306 individuals met inclusion criteria for the study cohort. Mean age at initial treatment was 63.5 years, sixty percent (60%) of the cohort patients were male. Thirty percent (30%, *n* = 8,373) of patients met the criteria for low-risk, i.e., < 60 years with no prior thrombotic events. Patients classified as low-risk were younger than patients classified as high-risk (mean age 49.0 vs. 69.6 for patients classified as high-risk) and had a higher proportion of males to females (low-risk: 2.3 times males:females, high-risk: 1.3 times males:females). PV treatment history was available for ≥ 1 year in 62% of patients and ≥ 3 years in 35% of patients. Table 1 summarizes the demographic and clinical characteristics of the full study cohort, stratified by risk status.

**Table 1:** Patient demographics and clinical characteristics at index date: full study cohort and by baseline patient risk group

	Study cohort	High-risk	Low-risk	*p* value^b^
Total	*N* = 28,306	*n* = 19,933	*n* = 8373	
Age, years, mean ± SD^a^	63.5 ± 12.7	69.6 ± 8.5	49.0 ± 8.7	< 0.0001
Age distribution, *n* (%)				< 0.0001
60 and over	18,496 (65)	18,496 (93)	(0)	
59 and under	9808 (35)	1436 (7)	8372 (100)	
Sex, *n* (%)				< 0.0001
Male	17,119 (60)	11,305 (57)	5814 (69)	
Female	11,187 (40)	8628 (43)	2559 (31)	
Race, *n* (%)				< 0.0001
White	18,085 (64)	13,243 (66)	4,842 (58)	
Unknown/other	6411 (23)	4,149 (21)	2,262 (27)	
Hispanic	1412 (5)	889 (4)	523 (6)	
Black	1382 (5)	951 (5)	431 (5)	
Mixed	728 (3)	518 (3)	210 (3)	
Asian	288 (1)	183 (1)	105 (1)	
Thrombotic event history, *n* (%)				
Prior thrombotic event	5587 (20)	5587 (28)	0 (0)	NA

^a^Patients age ≥ 80 and ≤ 18 were normalized to 80 and 18 years, respectively, for privacy reasons; age missing in 2 patients.

^b^*P* value is for the comparison of high-risk to low-risk, by chi-square or unequal variance two sample *t*-test

*NA*, not applicable; *SD*, standard deviation

**Fig. 2:**
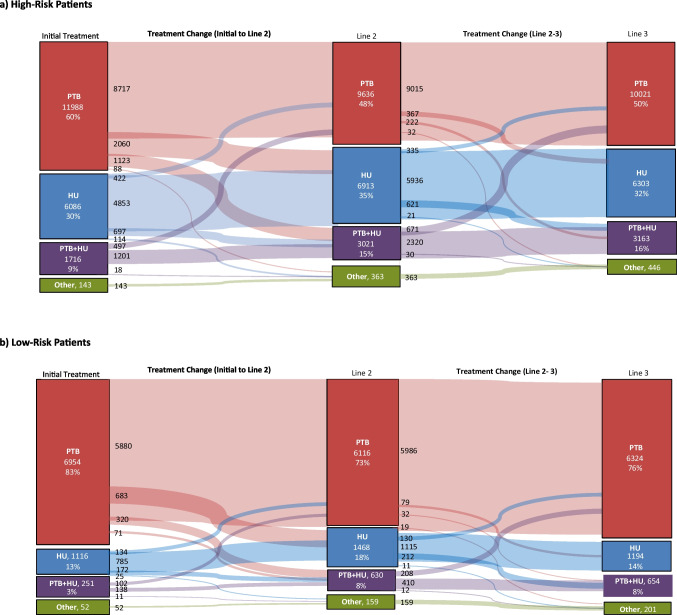
Patient treatment journey through up to 3 lines of therapy, by patient risk group

Analysis of treatment pathways indicated that a majority of patients, (18,942 [67%]), irrespective of risk status, started with phlebotomy monotherapy (Fig. 2). Twenty-five percent (7,202 [25%]) of patients started with hydroxyurea monotherapy; few patients (195 [1%]) had therapy other than phlebotomy and/or hydroxyurea as a first-line treatment. Of these, 116 (59%) had ruxolitinib phosphate alone or in combination, 75 (38%) had interferon alone or in combination, and 4 (2%) had busulfan. A small percentage of patients, 6% (1072/18,942), initiating treatment with phlebotomy monotherapy switched back to phlebotomy alone after adding or switching to hydroxyurea as second-line therapy.

Treatment was initiated with phlebotomy monotherapy in 60% of high-risk patients (Fig. 2a). (Of the 11,988 (60%) high-risk patients who started on phlebotomy monotherapy, 27% changed therapy, 63% switched to hydroxyurea, and 34% added hydroxyurea while continuing phlebotomy treatments. Of the 6086 high-risk patients (31%) who started on hydroxyurea monotherapy, 20% changed therapy, 34% switched to phlebotomy alone (i.e. discontinued hydroxyurea), while 57% added phlebotomy while continuing treatment with hydroxyurea. Overall, 74% of high-risk patients stayed on their first-line therapy throughout the study period.

Eighty-three percent (83%) of low-risk patients initiated treatment with phlebotomy monotherapy, most of whom (81% of all low-risk patients and 85% of those initiating on phlebotomy monotherapy) stayed on their first-line therapy throughout the study period (Fig. 2b). Of those who started on phlebotomy monotherapy and changed therapy (15%), 64% switched to hydroxyurea, and 30% added hydroxyurea while continuing phlebotomy treatments. Thirteen percent (13%) of low-risk patients initiated treatment with hydroxyurea monotherapy, of whom 70% retained that therapy throughout the study period. Among low-risk patients who started on hydroxyurea monotherapy and changed therapy (30%), 40% switched to phlebotomy, while 52% added phlebotomy while continuing treatment with hydroxyurea. Across all therapy lines, 81% of low-risk patients experienced no change in treatment regimen during the study.

Among patients in either risk group who had combination therapy with phlebotomy and hydroxyurea as a first- or second-line treatment and made a change, the overwhelming majority discontinued hydroxyurea.

### Evaluation of thrombotic event rates, by patient risk status

The occurrence of post-index thrombotic events was evaluated during a median period of 808 days. A total of 4549 (16%) individuals experienced at least one thrombotic event subsequent to treatment initiation, 20% (*n* = 3920) among patients classified as high-risk, and 8% (*n* = 629) among patients classified as low-risk. Forty percent (40%, *n* = 2,232) of patients with a documented prior thrombotic event experienced at least one thrombotic event subsequent to treatment initiation (Table 2). Stroke was the most commonly occurring thrombotic event (36% of total), followed closely by deep vein thrombosis (35%); 18% of patients experiencing a thrombotic event had a reported myocardial infarction (data not shown).

**Table 2:** Percent of patients experiencing thrombotic event (TE), by patient risk group

Risk group	Total patient count, *n*	Patients with TE post treatment initiation, *n* (%)
Low-risk patients		
Age < 60 and no prior TE	8373	629 (8)
High-risk patients		
All high-risk patients	19,933	3920 (20)
Patients with no prior TE	14,346	1688 (12)
Patients with prior TE	5587	2232 (40)

*TE*, thrombotic event

### Subgroup analysis of patients with HCT data

A subgroup of 4246 patients with outpatient laboratory data was evaluated, “HCT subgroup”. Mean age at initial treatment was 63.8 years; 2659 (62%) of the HCT subgroup patients were male (Online Resource 2). An evaluation of clinical and demographic characteristics at index indicated that the HCT subgroup was similar to the larger study cohort in age (63.5 vs. 63.8 years, *p* = 0.07), in the percentage of patients with prior thrombotic events (20% vs. 19%, *p* = 0.08), and in the proportion of high-risk patients (71% vs. 70%, respectively, *p* = 0.65) (Online Resource 2). Additionally, the treatment patterns of patients included in the HCT subgroup closely resembled those in the larger patient cohort, with 52% in each group receiving phlebotomy monotherapy, 20% in each group receiving hydroxyurea monotherapy, and 10% in each group switching from phlebotomy to hydroxyurea (Online Resource 3).

**Fig. 3:**
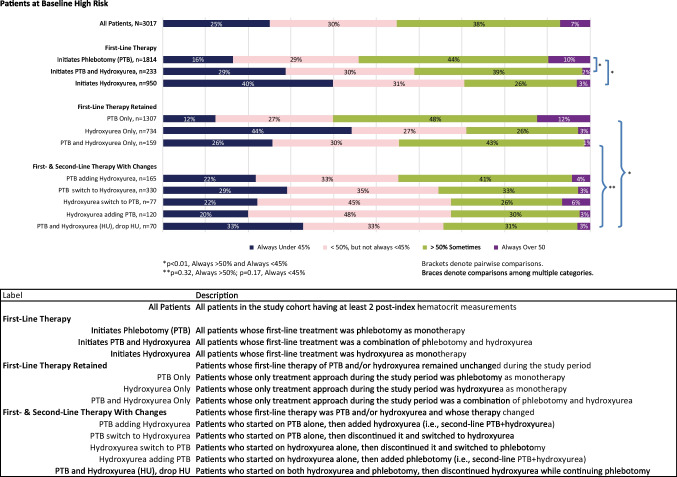
Hematocrit control, segmented by first-line treatment, and by first and second lines of treatment and changes, high-risk patients

Among high-risk patients, across all therapy lines, only 25% of patients had HCT levels consistently below 45%, while 7% had HCT values always > 50% (Fig. 3). High-risk patients initiating therapy with hydroxyurea with or without phlebotomy were more likely to meet clinical guidelines for HCT management (< 45%) compared to patients initiating phlebotomy monotherapy (29%, *p* = 0.002 and 40%, *p* < 0.0001, respectively, vs. 16% for phlebotomy monotherapy) (Fig. 3). High-risk patients initiating treatment with phlebotomy monotherapy were associated with the most unfavorable HCT results; 10% of high-risk patients initiating with phlebotomy monotherapy always had HCT levels greater than 50% compared to patients initiating with hydroxyurea monotherapy (3%, *p* < 0.0001) or phlebotomy and hydroxyurea combination (2%, *p* < 0.0001).

Despite suboptimal HCT management, phlebotomy monotherapy was retained for a large proportion of high-risk patients (43%; 1307/3017). Nearly half (48%) of this group had HCT levels sometimes greater than 50% and 12% consistently reported HCT levels greater than 50% during follow-up. High-risk patients who initiated and retained hydroxyurea monotherapy had the most favorable HCT results, 44% of these patients had HCT levels that were consistently lower than 45%, and only 3% had HCT levels always above 50%. Notably, while some patients did add a therapy (9%; 285/3017), relatively few high-risk patients switched or discontinued therapies during the outcome period (16%; 477/3017). Among those who did, patients who changed from a combination of phlebotomy and hydroxyurea to phlebotomy alone had the most favorable HCT results; yet, only 33% had HCT levels consistently below 45%.

**Fig. 4:**
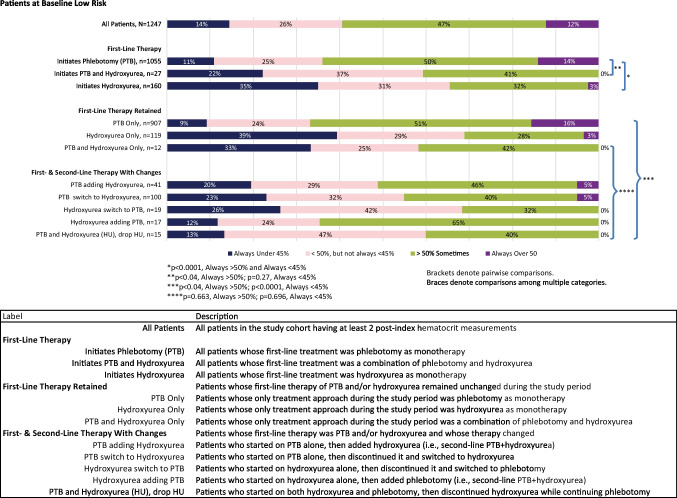
Hematocrit control, segmented by first-line treatment, and by first and second-lines of treatment and changes, low-risk patients

Among low-risk patients, across all therapy lines, 12% of patients had HCT levels consistently above 50%, and a total of 59% had some or all HCT tests above 50% (Fig. 4). Patients initiating treatment with phlebotomy monotherapy had the least favorable levels of HCT control; a total of 64% of patients sometimes (50%) or always (14%) had HCT levels above 50%. In comparison, low-risk patients treated with hydroxyurea with or without phlebotomy were less likely to have HCT levels consistently higher than 50%; 3% of low-risk patients treated with hydroxyurea monotherapy and 0% of patients treated with a combination of hydroxyurea and phlebotomy had HCT levels always above 50% (*p* < 0.0001 for both comparisons to phlebotomy monotherapy).

Among the 86% (907/1055) of low-risk patients retaining phlebotomy monotherapy, HCT results were the least favorable, with 16% consistently reporting HCT results greater than 50%. Low-risk patients initiating and retaining hydroxyurea monotherapy had consistently better HCT results than either phlebotomy monotherapy or phlebotomy and hydroxyurea combination, although the results with hydroxyurea treatment alone still showed suboptimal correspondence with clinical guidelines, i.e., 31% of patients had HCT results sometimes or always above 50%. Eleven percent (11%, 134/1247) of low-risk patients switched or discontinued therapies during the outcome period, the majority switching from phlebotomy monotherapy to hydroxyurea (75%, 100/134), yet 45% of this group had HCT levels sometimes or always above 50% (Fig. 4).

Pairwise comparisons in both high-risk and low-risk patient groups evaluating all therapy sequences involving both phlebotomy and hydroxyurea in any sequence or combination produced statistically similar results with respect to the proportions of patients with HCT levels consistently above 50% (*p* = 0.32 for high-risk group comparisons and *p* = 0.663 for low-risk patient groups) (Figs. 3 and 4).

Individuals for whom HCT results were available (HCT subgroup) experienced post-treatment thrombotic events at similar rates to the full study cohort (16.1% in each, *p* = 0.98). Eight percent (8%) of the low-risk group and 20% of the high-risk group experienced at least one thrombotic event (Online Resource 4). Stroke and DVTs were the most common thrombotic events at 2% each for the low-risk HCT subgroup and ranging from 3 to 20% for the high-risk HCT subgroup. In a subcohort of 3445 patients with up to 5 years of follow-up in medical claims data, 36% (236/653) of patients with a thrombotic event in their history experienced at least one other event. The most common events were deep vein thrombosis and stroke. Among the 397 high-risk patients who had another thrombotic event, 180 (45%) were treated with phlebotomy only and never switched to any other therapies.

## Discussion

To our knowledge, this is the largest published real-world study reporting treatment patterns and thrombotic event rates in PV patients of all ages and risk categories. The results obtained from this study provide important information about the current clinical management of PV in the USA.

Clinical guidelines for treatment of polycythemia vera have evolved somewhat over time [14, 15]. As the association of higher hematocrit levels and the risk of thrombotic events has been further elucidated [16–18], guidance regarding hematocrit control has become more important. Our study shows a significant gap between recommended treatment and actual treatment patterns in a community-dwelling population. In our study, the majority of high-risk patients (60%) were managed with phlebotomy monotherapy as first-line treatment (while hydroxyurea monotherapy as first-line treatment was used in 30% of high-risk patients). These results show a considerably higher rate of hydroxyurea use compared to a recent large study by Pemmaraju et al. (*N* = 50,405 Medicare beneficiaries with PV, all high-risk), which reported a 12% rate of hydroxyurea treatment [19]. An analysis of clinical and disease characteristics at baseline from the REVEAL study of 2510 patients with PV and similar characteristics (mean age of 66.3, 77.3% high-risk), to our study’s overall patient population (mean age of 63.5, 70.6% high-risk), estimated that 33.6% of patients were treated with phlebotomy alone, 29.0% with hydroxyurea alone, and 23.7% with both phlebotomy and hydroxyurea [20].

In our study, 74% of high-risk patients stayed on their first-line therapy throughout the study period. Among the 27% of high-risk patients who started on phlebotomy monotherapy and switched, 63% switched to hydroxyurea and 34% added hydroxyurea while continuing phlebotomy treatments. As second- and third-line treatment, hydroxyurea was prescribed to 50% and 48% of high-risk patients, respectively. Among the low-risk patients who started on phlebotomy monotherapy and switched, 64% switched to hydroxyurea and 30% added hydroxyurea while continuing phlebotomy treatments. Hydroxyurea was prescribed as second- and third-line therapies in 26% and 22% of low-risk patients, respectively.

Our subgroup analysis included 4246 PV patients with HCT measurements and characteristics similar to those of the entire cohort in terms of age, percentage of patients with prior thrombotic events, proportion of high-risk patients, and patterns of clinical management. Only 25% of patients in the high-risk group had all HCT measurements < 45% in the 2-year follow-up period, whereas 7% of patients in the high-risk group were very poorly controlled with all HCT measurements above 50%, and an additional 38% of patients had some HCT tests > 50%. These results indicate that, despite treatment, the majority of high-risk patients were poorly controlled. The percentage of high-risk patients who did not meet the NCCN guideline target of HCT < 45% in our study (75%) is considerably higher than the 42.9% rate reported in the REVEAL study, a difference that could be attributed to the prospective nature of the REVEAL study which required physician referral to the study and the fact that all patients had been treated with hydroxyurea for a minimum of 3 months [21]. Further analysis of our study results by treatment mode showed that regardless of treatment received, a sizable proportion of patients (ranging from 29% of high-risk patients taking hydroxyurea as first-line treatment, to 67% of low-risk patients treated with phlebotomy as first-line treatment) had at least one hematocrit measurement above 50% in the study period. Our study thus confirms and reinforces the findings of the REVEAL study, according to which a significant percentage of both high- and low-risk PV patients do not reach the NCCN target of HCT < 45%.

PV patients are at higher risk of thrombotic complications which are the main cause of mortality [22] and are associated with higher resource utilization and healthcare costs [23]. A retrospective study of 1322 PV patients identified in private insurance data by Parasuraman et al. (2018) reported that 16.3% of patients experienced thromboembolic events in the 112-month follow-up period following treatment initiation [23]. A more recent retrospective observational study by Pemmaraju et al. (2022) analyzed Medicare health claims of 50,405 patients with PV and reported a higher rate (28.4%) of thrombotic events during a median follow-up period of 34.5 months, with most commonly reported TE events being ischemic stroke (46.0%), transient ischemic attack (30.7%), and acute myocardial infraction (29.9%) [19]. The higher rate of thrombotic event observed in the Pemmaraju (2022) study may be related to the longer follow-up period of the study and the higher median age (73 years) of study subjects.

Consistent with these two recently published studies, thrombotic events were not uncommon in our study, particularly among patients classified as high-risk. During a median evaluation period of 808 days (2.2 years), at least one thrombotic event subsequent to treatment initiation was reported in 16% of patients overall (20% of high-risk and 8% of low-risk patients), with stroke (36%), deep vein thrombosis (35%), and myocardial infarction (18%) being the most frequently reported TEs. Our study results are in line with prior research findings which indicate that PV patients experience a high rate of thrombotic events irrespective of treatment pathway, highlighting the need to improve patient management, even though our study design does not establish associations between specific treatment pathways and the risk of thrombotic events.

There are certain limitations of this study that are consistent with the retrospective nature of claims-based analyses and include the potential for incomplete or missing records and misdiagnosis of PV, as diagnoses were based solely on database records. There is also the possibility that thrombotic events were misdiagnosed, under-diagnosed, or otherwise, incorrectly reported. Since the dominant data source of this study is private insurance claims, uninsured patients are not included, and patients insured by Medicaid or fee-for-service Medicare are likely to be under-represented. Some of the treatments for PV are dispensed from specialty pharmacies, e.g., ruxolitinib, which may not be fully captured in the Symphony data, so the utilization of these therapies may be under-reported. Additionally, aspirin is often recommended to patients without prescription so the available pharmaceutical data was not considered a reliable source to assess aspirin use, nor did we attempt to associate the use of other anti-thrombotic medications with the observed rates of thrombotic events. Hematocrit data was available on a limited subgroup of the population, although a comparison of baseline characteristics showed similarity between the subgroup and the larger patient cohort. Further, the laboratory values were limited to a single-source outpatient laboratory provider. While it is reasonable to hypothesize that patients are likely to use the same commercial laboratory provider for repeated outpatient tests, tests run at other laboratories (including any inpatient or outpatient hospital laboratory tests) would not be included, and the hypothesis cannot be tested. The groups of patients receiving different combinations and sequences of therapies are not matched cohorts, and results on hematocrit testing displayed in Figs. 3 and 4 were not segmented before and after therapy changes. Therapies are changed for many reasons, including lack of tolerance or compliance, side effects, inadequate results, and cost. However, when treatment is initiated on one therapy and a second therapy is added to it, the dissatisfaction with results of the initial therapy alone is the most logical rationale. Consistent with this theory, we note that patients who started on hydroxyurea and added phlebotomy had fewer HCT tests always under 45% compared to patients managed on hydroxyurea alone (23% vs. 44%, respectively). In contrast, patients who were initiated with phlebotomy and subsequently added hydroxyurea had higher overall levels of guideline-compliance compared to patients managed on phlebotomy alone (21% vs. 14%). In fact, the most common therapeutic approach, phlebotomy alone, was associated with the lowest level of guideline-compliant results (14%) of all the therapeutic pathways.

## Conclusion

These descriptive findings merit more detailed exploration in other studies, but these data do indicate substantial collective reliance on an established therapy, phlebotomy, despite a low percentage of patients achieving guideline-recommended results in hematocrit control, and 8% of low-risk and 20% of high-risk patients experiencing thrombotic events despite access to all available treatment options.

## Supplementary Information

Below is the link to the electronic supplementary material.

Supplementary file1 (PDF 262 KB)

## Data Availability

The dataset supporting the conclusions in this article is available from Symphony Health Solutions IDV®. However, restrictions apply to the availability of these data, which were used under license for the current study and so are not publicly available.

## Code availability

Diagnosis and procedure codes (as used) are listed in Supplement Table 1.
