# Supplementary material for: Real-world treatments and thrombotic events in polycythemia vera patients in the USA
Source: Ann Hematol. 2023 Jan 13;102(3):571–81. doi: 10.1007/s00277-023-05089-6 (PMC9977710; doi:10.1007/s00277-023-05089-6)
Supplement: Supplementary file 1 — Supplementary file1 (PDF 262 KB) [file 277_2023_5089_MOESM1_ESM.pdf]

Electronic Supplemental Material

Title: Real World Treatments and Thrombotic Events in Polycythemia Vera Patients in the USA

Journal: Annals of Hematology

Authors: SrdanVerstovsek[1], Naveen Pemmaraju[1], Nancy L Reaven[2], Susan E Funk[2], Tracy Woody[3], Frank Valone[3], Suneel Gupta[3]

[1]MDAnderson Cancer Center, Houston, TX, [2]Strategic Health Resources, La Canada CA, [3]Protagonist Therapeutics, Newark, CA.

Corresponding author: Nancy L Reaven, Strategic Health Resources, nancy.reaven@strathealth.com

Supplement Table 1. Sources and Methods

Supplement Table 2. Patient Characteristics, HCT Subgroup

Supplement Table 3. Treatment Pattern (First and Second Therapy Lines) for Study Cohort and HCT Subgroup

Supplement Table 4. Thrombotic Events (TE) Post Treatment Initiation in Patients in HCT Subgroup

Supplement Table 1. Sources and Methods

Variable	Definition	Methodology
**Selection Criteria**		
Polycythemia Vera (PV) diagnosis	ICD-9 238.4 or ICD-10 D45	One or more occurrences of a diagnosis code in any setting of care.
Treatment for PV	Phlebotomy, hydroxyurea, interferon alfa 2-a, interferon alfa 2-b, ruxolitinib phosphate, or other (see below)	One or more occurrences of filled prescription, CPT® or HCPCS code in any setting of care; "Other" is identified by procedure code but requires confirming evidence of either a qualifying drug listed on the procedure claim or for the patient to have a diagnosis of interest within the index period.
**Treatment for PV by Type**	**Drug Names**	**Procedure codes**
Phlebotomy		99195
Hydroxyurea	Droxia®, Hydrea®, hydroxyurea, Myocel®, or Siklos®	S0176
Interferons and Jakafi®	Interferon alfa-2a, recombinant (Roferon-A®), interferon alfa-2b, recombinant (Intron A®), peginterferon alfa-2a (Pegasys®, Pegasys® Proclick®), peginterferon alfa-2b (Pegintron®, Pegintron® Redipen®, Sylatron®, Sylatron® 4-Pack), ruxolitinib phosphate (Jakafi®)	J9213, J9214, S0145, S0148, S0146
Other		J3590 (unclassified biologics) or J8999 (prescription drug, oral, chemotherapeutic, NOS)

Demographics		
Age	Age at time of treatment	Age was established as of the initial treatment for PV.
Race	Race and ethnicity, combined from separate variables	Race and ethnicity were obtained from a combination of sources using proprietary methods prior to data de-identification.
**Lines of Therapy**	**Description**	**Definition**
First line therapy	The single product or combination of products first observed for a patient.	The combination of treatments occurring within 30 days of index and -- for pharmaceutical agents -- repeated within the first 60 days post-index.
Additional of a new product	When a claim occurs for any new treatment or combination of treatments within market basket*. Phlebotomy claim should be within the days of supply of previous HU claim + 30 days of grace period.*	A new treatment occurring within Days Supply + a 30-day grace period of the prior treatment, where the prior treatment is repeated within Days Supply + 30 days of the added treatment.
Product switch	When claim occurs for any new treatment or combination of treatments within market basket.	A new treatment occurring, where the prior treatment is not repeated within Days Supply + 30 days of the added treatment.
Product discontinuation	When one (or multiple) product was discontinued from current regimen.	When a treatment previously identified is not repeated within Days Supply + 30, and available data does not end within Days Supply + 30 of the last occurrence.
Product restart	When one (or multiple) product was discontinued from current regimen and added back later.	When a treatment that has met the criteria above for discontinuation subsequent recurs in claims.
Days supply		Days supply specified in the pharmacy claim; zero for a phlebotomy procedure.

Thrombotic Event (Outcome)		
Thrombotic event (outcome)	Abdominal Thrombosis, Myocardial Infarction (MI), Transient Ischemic Attack (TIA), Stroke, Pulmonary Embolism (PE), Peripheral Arterial Thrombosis, Deep Vein Thrombosis (DVT), or Acute Coronary Syndrome.	Defined by single occurrence of any relevant diagnosis code in medical claims data from all settings of care between treatment initiation and March 2020. Code ranges shown below; header codes (invalid) not included.
History of thrombotic event (TE)	Recorded TE events within a year prior to treatment initiation.	Defined by single occurrence of any relevant diagnosis code in medical claims data from all settings of care during the year prior to but not including the date of treatment initiation. Code ranges shown below; header codes (invalid) not included.
**Type of Thrombotic Event**	**ICD-9-CM codes**	**ICD-10-CM codes**
Acute coronary syndrome	411, 411.8, 411.89	I24.0, I24.8, I24.9
Abdominal thrombosis	444.0-444.09; 557.0-557.9	I74.01; I74.09; K55.0-K55.069
Deep vein thrombosis (DVT)	451.83; 453.41-453.42; 453.82	I82.401-I82.409; I82.491-I82.5Z9; I82.621-I82.629; I82.721-I82.729
Myocardial infarction (MI)	410.0-410.92	I21.A1, I21.A9, I21.0-I21.9
Other thrombosis	362.34; 444.1; 444.1; 444.81-444.9	H34.10-H34.13; I74.10-I74.19; I74.5-I74.9

Peripheral arterial thrombosis	444.21-444.22; 453.8-453.81; 453.83-453.89	I74.2-I74.4; I82.601-I82.619; I82.811-I82.819
Pulmonary embolism (PE)	451.1-415.19	I26.01-I26.09, I26.90-I26.99, I27.82
Stroke	430-431; 433.01; 433.11; 433.21; 433.31; 433.81; 433.91; 434.01; 434.11; 434.91	I60.00-I61.9; I63.00-I63.9
Transient ischemic attack (TIA)	435.8-435.9;	G45.8-G45.9

Supplement Table 2. Patient Characteristics, HCT Subgroup

	**Total HCT Subgroup**	**High-Risk**	**Low-Risk**	***P* value**[b]	***P* value, Subgroup vs Full Cohort**
Total	*n*=4,264	*n*=3,017	*n*=1,247		0.0803
Age, years, mean ± SD[a]	63.8 ± 12.0	69.6 ± 8.0	50.0 ± 8.2	<0.0001	0.073
Age Distribution, *n* (%)				<0.0001	0.425
59 and under	1,451 (34)	204 (7)	1,247 (100)		
60 and over	2,813 (66)	2,813 (93)	(0)		
Sex, *n* (%)				<0.0001	0.019
Female	1,605 (38)	1,241 (41)	364 (29)		
Male	2,659 (62)	1,776 (59)	883 (71)		
Race, *n* (%)				<0.0001	<0.0001
Asian	60 (1)	35 (1)	25 (2)		
Black	181 (4)	124 (4)	57 (5)		
Hispanic	319 (7)	210 (7)	109 (9)		
Mixed	119 (3)	77 (3)	42 (3)		
White	2,632 (62)	1,953 (65)	679 (54)		
Unknown/Other	953 (22)	618 (20)	335 (27)		
Thrombotic Event History, *n* (%)					0.080
Prior TE	793 (19)	793 (26)	0 (0)		

[a]Patients age ≥80 and ≤18 were normalized to 80 and 18 years, respectively, for privacy reasons.
[b]*P* value is for the comparison of high risk to low risk, by Chi-square or unequal variance two sample t-test.

HCT=hematocrit; SD=standard deviation; TE=thrombotic event.

Supplement Table 3. Treatment Pattern (First and Second Therapy Lines) for Study Cohort and HCT Subgroup

	**HCT Subgroup, *n* (%)**	**Study Cohort, *n* (%)**
Hydroxyurea only	853 (20)	5638 (20)
PTB & Hydroxyurea, dropping Hydroxyurea	85 (2)	599 (2)
PTB Δ to Hydroxyurea	430 (10)	2743 (10)
PTB & Hydroxyurea only	171 (4)	1339 (5)
Hydroxyurea, adding PTB	96 (2)	869 (3)
PTB, adding Hydroxyurea	206 (5)	1443 (5)
Hydroxyurea Δ to PTB	137 (3)	556 (2)
PTB only	2214 (52)	14597 (52)
Subtotal: most common therapies	4192 (98)	27784 (98)
All others	72 (2)	522 (2)
Grand total	4264 (100)	28306 (100)

HCT=hematocrit; PTB=phlebotomy.

Note: Total of first-line therapies does not correspond to Figure 2 because patients because patients starting with phlebotomy and/or Hydroxyurea and having some other therapy as line 2 are grouped into “all others” above.

Supplement Table 4. Thrombotic Events (TE) Post Treatment Initiation in Patients in HCT Subgroup

Risk Group	Total Patient Count, *n*	Patient With TE Post Treatment Initiation, *n* (%)
Low-Risk Patients		
Age <60 and no prior TE	1,247	95 (8)
High-Risk Patients		
All high-risk patients	3,017	591 (20)
Patients with no prior TE	2,224	262 (12)
Patients with prior TE	793	329 (41)

HCT=hematocrit; TE=thrombotic event.
